# Supplementary material for: Identification of Glyceraldehyde-3-Phosphate Dehydrogenase Gene as an Alternative Safe Harbor Locus in Pig Genome
Source: Genes (Basel). 2019 Aug 29;10(9):660. doi: 10.3390/genes10090660 (PMC6770653; doi:10.3390/genes10090660)
Supplement: Supplementary file 1 [file genes-10-00660-s001.zip › genes-571308-supplementary-revised/Supplementary Materials.docx]

**Figure S1**


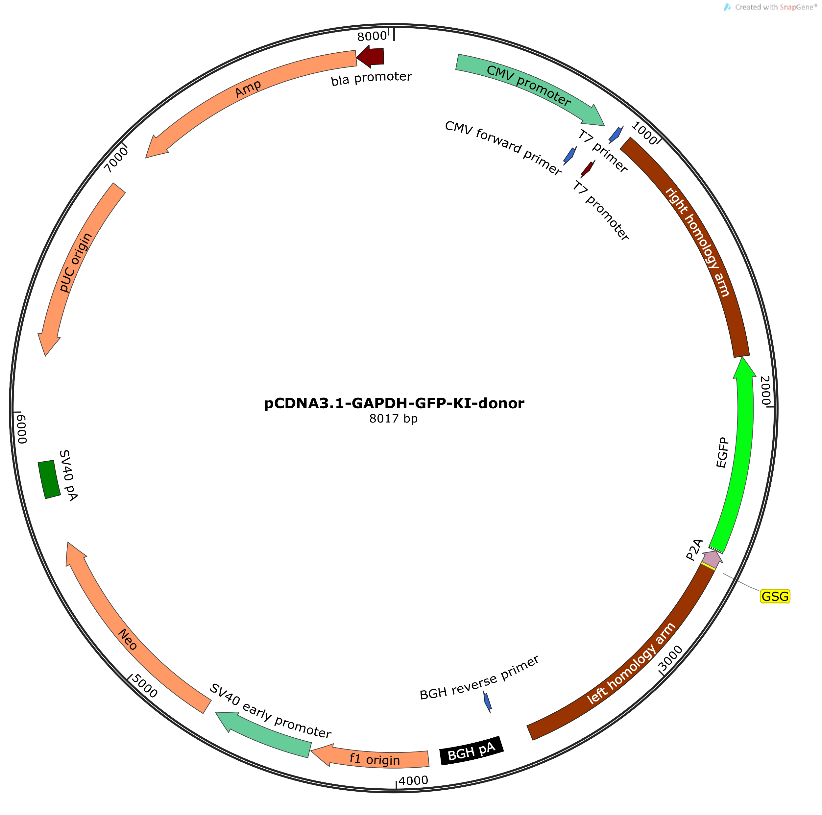


**Figure S1.** Vector map of donor vector (Pcdna3.1-GAPDH-GFP-KI-donor). The P2A-GFP fragment flanking with homology arms were synthesized and inserted reversed into pCDNA3.1+ by restriction enzymes.

**Detailed donor vector sequences**

The sequences of homolog arms and P2A-GFP fragments are marked in different colors corresponding to the vector map mentioned above.

gacggatcgggagatctcccgatcccctatggtgcactctcagtacaatctgctctgatgccgcatagttaagccagtatctgctccctgcttgtgtgttggaggtcgctgagtagtgcgcgagcaaaatttaagctacaacaaggcaaggcttgaccgacaattgcatgaagaatctgcttagggttaggcgttttgcgctgcttcgcgatgtacgggccagatatacgcgttgacattgattattgactagttattaatagtaatcaattacggggtcattagttcatagcccatatatggagttccgcgttacataacttacggtaaatggcccgcctggctgaccgcccaacgacccccgcccattgacgtcaataatgacgtatgttcccatagtaacgccaatagggactttccattgacgtcaatgggtggagtatttacggtaaactgcccacttggcagtacatcaagtgtatcatatgccaagtacgccccctattgacgtcaatgacggtaaatggcccgcctggcattatgcccagtacatgaccttatgggactttcctacttggcagtacatctacgtattagtcatcgctattaccatggtgatgcggttttggcagtacatcaatgggcgtggatagcggtttgactcacggggatttccaagtctccaccccattgacgtcaatgggagtttgttttggcaccaaaatcaacgggactttccaaaatgtcgtaacaactccgccccattgacgcaaatgggcggtaggcgtgtacggtgggaggtctatataagcagagctctctggctaactagagaacccactgcttactggcttatcgaaattaatacgactcactatagggagacccaagctggctagcgtttaaacttaagcttTGGAATCTAATCCTAATGAAACTGGCGAGTATTTATTGAACGCTTTGGTGTCACTGTTTCCAGCAACTTTTCGGTGGTGGCTCTGGTCCCTGTTGTGTGGTGTGCACTGGTGATCATTGCACAGACACACAGGGTTACAAGCAACTTGCCCAAAGTAATGGCACCTCAAGTAGCAGAACCAGGGCCTCCATCCAGGTTGTCAGTGCTGTCTCTGCTTCTTCCCACTAAGCCTGTGCGCATTCGCAGGTGACAGAAGACATGGAAATAAAACCACCCCAACCTTTCCCTTGGGCTAACCTACTGTAACTTCTCTGAACAATCTCTGAGCTGGCACTGGCACAGCTGTACCACAGATGGCAATCTCTTGGGCACTGAGGCTCACAGCTGTGACAGGGCAGAGCTGGAATCCACTCCTGGCAGTCAGGCCGTAGCCAAATGGACTTCCTGTGCCAGTGCCCCCCTCCAGGGAGCTGCAGACAGAATGGTGTCTTGGGTGCAAGCAGCCAGGCCATTCCTGGGTTCAGCCACGGCAGTCTGGAGCTGTAGCACCCAGAGGCCTCACTAAGGCAACACGAGGACAGGTGGCTCTTCAGAGGAGCCCAGGGTGGAGTTTCCTCAGCAAGCTCTAGTTCTCCTTGGCCCCAGCCCGGCTTCCCTCCTCTTTATCCCAGGCTCTAGAGTGAGAACGACAGGAGGCTGGGTACAGTGTACTTTATTGATGGTACATGACGAGGCAGGTCTCCCTAAGCCCCTCCCCTTCTTCCGGGGGTCTGGGATGGAAACTGGAAGTCAGGAGATGCTCGGTGTGTTGGGGGATCGAGTTGGGGCTGTGACTCCCCAGCAGCTGAGGGCCTCTCTCCTCCTCGCGTGCTCTTGCTGGGGTTGGTGGTCCAGGGGCTCTTACTTGTACAGCTCGTCCATGCCGAGAGTGATCCCGGCGGCGGTCACGAACTCCAGCAGGACCATGTGATCGCGCTTCTCGTTGGGGTCTTTGCTCAGGGCGGACTGGGTGCTCAGGTAGTGGTTGTCGGGCAGCAGCACGGGGCCGTCGCCGATGGGGGTGTTCTGCTGGTAGTGGTCGGCGAGCTGCACGCTGCCGTCCTCGATGTTGTGGCGGATCTTGAAGTTCACCTTGATGCCGTTCTTCTGCTTGTCGGCCATGATATAGACGTTGTGGCTGTTGTAGTTGTACTCCAGCTTGTGCCCCAGGATGTTGCCGTCCTCCTTGAAGTCGATGCCCTTCAGCTCGATGCGGTTCACCAGGGTGTCGCCCTCGAACTTCACCTCGGCGCGGGTCTTGTAGTTGCCGTCGTCCTTGAAGAAGATGGTGCGCTCCTGGACGTAGCCTTCGGGCATGGCGGACTTGAAGAAGTCGTGCTGCTTCATGTGGTCGGGGTAGCGGCTGAAGCACTGCACGCCGTAGGTCAGGGTGGTCACGAGGGTGGGCCAGGGCACGGGCAGCTTGCCGGTGGTGCAGATGAACTTCAGGGTCAGCTTGCCGTAGGTGGCATCGCCCTCGCCCTCGCCGGACACGCTGAACTTGTGGCCGTTTACGTCGCCGTCCAGCTCGACCAGGATGGGCACCACCCCGGTGAACAGCTCCTCGCCCTTGCTCACCATGGGGCCGGGGTTCTCCTCCACGTCGCCGGCCTGCTTCAGCAGGCTGAAGTTGGTGGCTCCGCTTCCCTCTTTGGAGGCCATGTGGACCATGAGGTCCACCACCCTGTTGCTGTAGCCAAATTCATTGTCGTACCTGAAAGGAGAGCAAGAGTTAAGTGTGGTTTTCCCGAGCAGCCACCAGGGGGCACCAGACTTGATCACATTCTGAAGCATCATCCCTCCCCACCCGCCACATCCCCCAGCACCCTACCAGGAAATGAGCTTGACGAAGTGGTCGTTGAGGGCAATGCCAGCCCCAGCATCAAAAGTGGAAGAGTGAGTGTCACTGTTGAAGTCACAGGACACAACCTGGGGAAGGAAGAGGGCCAATTACGGAACCCAGTCTTGGTCAGTGGGGTCGTGTGGACACCAAGTTCCCTGACCTATCAGTACCTGGTCCTCAGTGTAGCCCAGGATGCCCTTGAGGGGGCCCTCGGACGCCTGCTTCACCACCTTCTTGATGTCATCATATTTTGCCTGATGAGGTAAAAACCGATGGTTATTCAAGTACCAATCCTACAACCCCTCAGCCCCCCAAAGCTGTTCCAACCCAAACCTACAGGTTTCTCCAGGCGGCAGGTCAGATCCACAACCGACACGTTGGGGGTGGGGACACGGAAGGCCATGCCAGTGAGCTTCCTGGGGACACGAGGAAGCAAGCAGAGTCAGCACCACCCCTGCTGGTGCCCCAGACACCCAGGGCAGGAGAGCAGCAAAGAGCCTCACCCGTTGAGCTCAGGGATGACCTTGCCCACAGCCTTGGCAGCGCCGGTAGAAGCAGGGATGATGTTCTGGGCAGCCCCTCGGCCATCACGCCACAGCTTCCCAGACGGGCCATCCACAGTCTTCTGGGTGGCAGTGATGGCATGGACCGTGGTCTGGAGACAAGGAGGGGGAGTGAAGGCCCTGCCTAGCGCTTGTGACGCCggtaccgagctcggatccactagtccagtgtggtggaattctgcagatatccagcacagtggcggccgctcgagtctagagggcccgtttaaacccgctgatcagcctcgactgtgccttctagttgccagccatctgttgtttgcccctcccccgtgccttccttgaccctggaaggtgccactcccactgtcctttcctaataaaatgaggaaattgcatcgcattgtctgagtaggtgtcattctattctggggggtggggtggggcaggacagcaagggggaggattgggaagacaatagcaggcatgctggggatgcggtgggctctatggcttctgaggcggaaagaaccagctggggctctagggggtatccccacgcgccctgtagcggcgcattaagcgcggcgggtgtggtggttacgcgcagcgtgaccgctacacttgccagcgccctagcgcccgctcctttcgctttcttcccttcctttctcgccacgttcgccggctttccccgtcaagctctaaatcgggggctccctttagggttccgatttagtgctttacggcacctcgaccccaaaaaacttgattagggtgatggttcacgtagtgggccatcgccctgatagacggtttttcgccctttgacgttggagtccacgttctttaatagtggactcttgttccaaactggaacaacactcaaccctatctcggtctattcttttgatttataagggattttgccgatttcggcctattggttaaaaaatgagctgatttaacaaaaatttaacgcgaattaattctgtggaatgtgtgtcagttagggtgtggaaagtccccaggctccccagcaggcagaagtatgcaaagcatgcatctcaattagtcagcaaccaggtgtggaaagtccccaggctccccagcaggcagaagtatgcaaagcatgcatctcaattagtcagcaaccatagtcccgcccctaactccgcccatcccgcccctaactccgcccagttccgcccattctccgccccatggctgactaattttttttatttatgcagaggccgaggccgcctctgcctctgagctattccagaagtagtgaggaggcttttttggaggcctaggcttttgcaaaaagctcccgggagcttgtatatccattttcggatctgatcaagagacaggatgaggatcgtttcgcatgattgaacaagatggattgcacgcaggttctccggccgcttgggtggagaggctattcggctatgactgggcacaacagacaatcggctgctctgatgccgccgtgttccggctgtcagcgcaggggcgcccggttctttttgtcaagaccgacctgtccggtgccctgaatgaactgcaggacgaggcagcgcggctatcgtggctggccacgacgggcgttccttgcgcagctgtgctcgacgttgtcactgaagcgggaagggactggctgctattgggcgaagtgccggggcaggatctcctgtcatctcaccttgctcctgccgagaaagtatccatcatggctgatgcaatgcggcggctgcatacgcttgatccggctacctgcccattcgaccaccaagcgaaacatcgcatcgagcgagcacgtactcggatggaagccggtcttgtcgatcaggatgatctggacgaagagcatcaggggctcgcgccagccgaactgttcgccaggctcaaggcgcgcatgcccgacggcgaggatctcgtcgtgacccatggcgatgcctgcttgccgaatatcatggtggaaaatggccgcttttctggattcatcgactgtggccggctgggtgtggcggaccgctatcaggacatagcgttggctacccgtgatattgctgaagagcttggcggcgaatgggctgaccgcttcctcgtgctttacggtatcgccgctcccgattcgcagcgcatcgccttctatcgccttcttgacgagttcttctgagcgggactctggggttcgaaatgaccgaccaagcgacgcccaacctgccatcacgagatttcgattccaccgccgccttctatgaaaggttgggcttcggaatcgttttccgggacgccggctggatgatcctccagcgcggggatctcatgctggagttcttcgcccaccccaacttgtttattgcagcttataatggttacaaataaagcaatagcatcacaaatttcacaaataaagcatttttttcactgcattctagttgtggtttgtccaaactcatcaatgtatcttatcatgtctgtataccgtcgacctctagctagagcttggcgtaatcatggtcatagctgtttcctgtgtgaaattgttatccgctcacaattccacacaacatacgagccggaagcataaagtgtaaagcctggggtgcctaatgagtgagctaactcacattaattgcgttgcgctcactgcccgctttccagtcgggaaacctgtcgtgccagctgcattaatgaatcggccaacgcgcggggagaggcggtttgcgtattgggcgctcttccgcttcctcgctcactgactcgctgcgctcggtcgttcggctgcggcgagcggtatcagctcactcaaaggcggtaatacggttatccacagaatcaggggataacgcaggaaagaacatgtgagcaaaaggccagcaaaaggccaggaaccgtaaaaaggccgcgttgctggcgtttttccataggctccgcccccctgacgagcatcacaaaaatcgacgctcaagtcagaggtggcgaaacccgacaggactataaagataccaggcgtttccccctggaagctccctcgtgcgctctcctgttccgaccctgccgcttaccggatacctgtccgcctttctcccttcgggaagcgtggcgctttctcatagctcacgctgtaggtatctcagttcggtgtaggtcgttcgctccaagctgggctgtgtgcacgaaccccccgttcagcccgaccgctgcgccttatccggtaactatcgtcttgagtccaacccggtaagacacgacttatcgccactggcagcagccactggtaacaggattagcagagcgaggtatgtaggcggtgctacagagttcttgaagtggtggcctaactacggctacactagaagaacagtatttggtatctgcgctctgctgaagccagttaccttcggaaaaagagttggtagctcttgatccggcaaacaaaccaccgctggtagcggtggtttttttgtttgcaagcagcagattacgcgcagaaaaaaaggatctcaagaagatcctttgatcttttctacggggtctgacgctcagtggaacgaaaactcacgttaagggattttggtcatgagattatcaaaaaggatcttcacctagatccttttaaattaaaaatgaagttttaaatcaatctaaagtatatatgagtaaacttggtctgacagttaccaatgcttaatcagtgaggcacctatctcagcgatctgtctatttcgttcatccatagttgcctgactccccgtcgtgtagataactacgatacgggagggcttaccatctggccccagtgctgcaatgataccgcgagacccacgctcaccggctccagatttatcagcaataaaccagccagccggaagggccgagcgcagaagtggtcctgcaactttatccgcctccatccagtctattaattgttgccgggaagctagagtaagtagttcgccagttaatagtttgcgcaacgttgttgccattgctacaggcatcgtggtgtcacgctcgtcgtttggtatggcttcattcagctccggttcccaacgatcaaggcgagttacatgatcccccatgttgtgcaaaaaagcggttagctccttcggtcctccgatcgttgtcagaagtaagttggccgcagtgttatcactcatggttatggcagcactgcataattctcttactgtcatgccatccgtaagatgcttttctgtgactggtgagtactcaaccaagtcattctgagaatagtgtatgcggcgaccgagttgctcttgcccggcgtcaatacgggataataccgcgccacatagcagaactttaaaagtgctcatcattggaaaacgttcttcggggcgaaaactctcaaggatcttaccgctgttgagatccagttcgatgtaacccactcgtgcacccaactgatcttcagcatcttttactttcaccagcgtttctgggtgagcaaaaacaggaaggcaaaatgccgcaaaaaagggaataagggcgacacggaaatgttgaatactcatactcttcctttttcaatattattgaagcatttatcagggttattgtctcatgagcggatacatatttgaatgtatttagaaaaataaacaaataggggttccgcgcacatttccccgaaaagtgccacctgacgtc

**Figure S2**

**
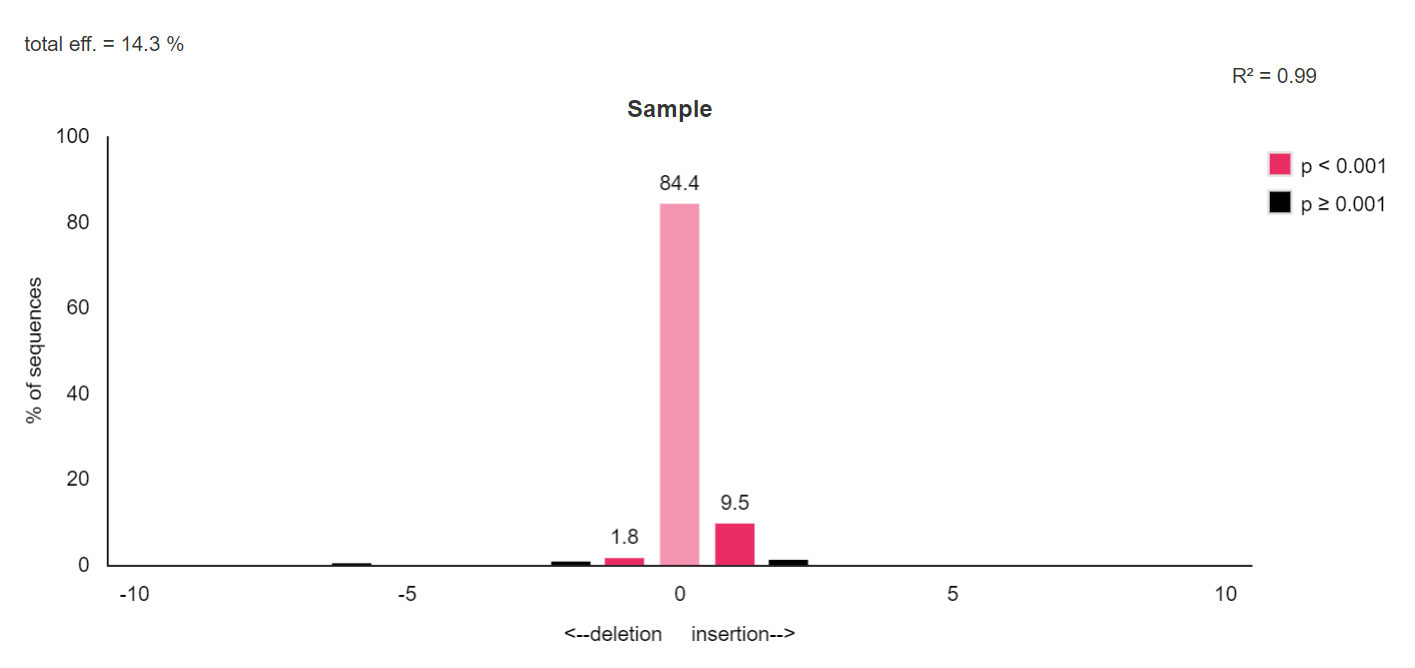
**

**Figure S2.** TIDE analysis of Indel rate of the GAPDH-sgRNA. The Indel rate of GAPDH-sgRNA is shown at the upper left corner.

**Figure S3**

**
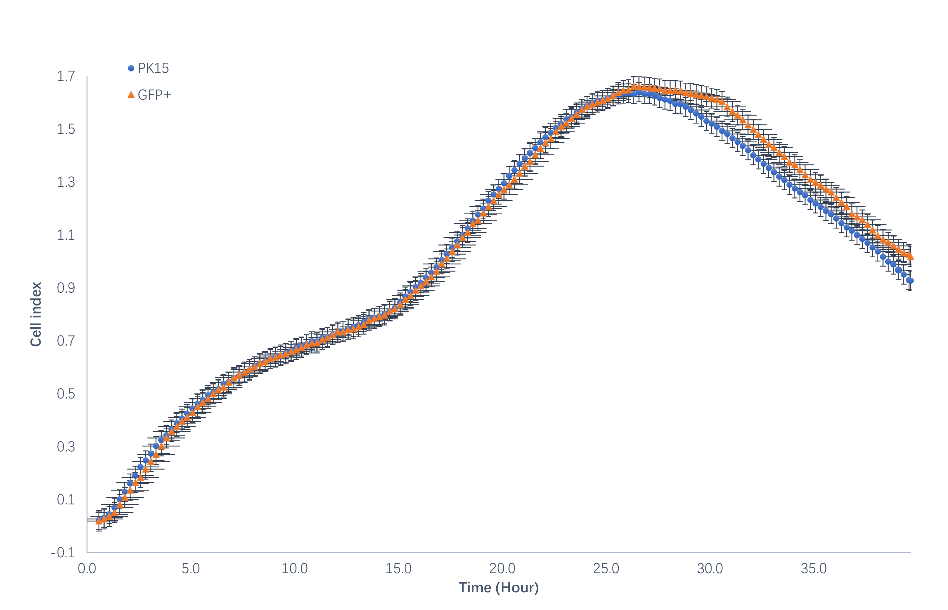
**

**Figure S3.** The growth curves of PK15 cells and GFP+ PK15cells. Cells were seeded at a concentration of 3500 cells/well and the average cell index at each time point was plotted against time. The data represent the means of three independent experiments. Error bars indicate the standard deviations of three experiments. PK15, wild type PK15 cells; GFP+, GFP knock-in PK15 cells.

**Figure S4**

**
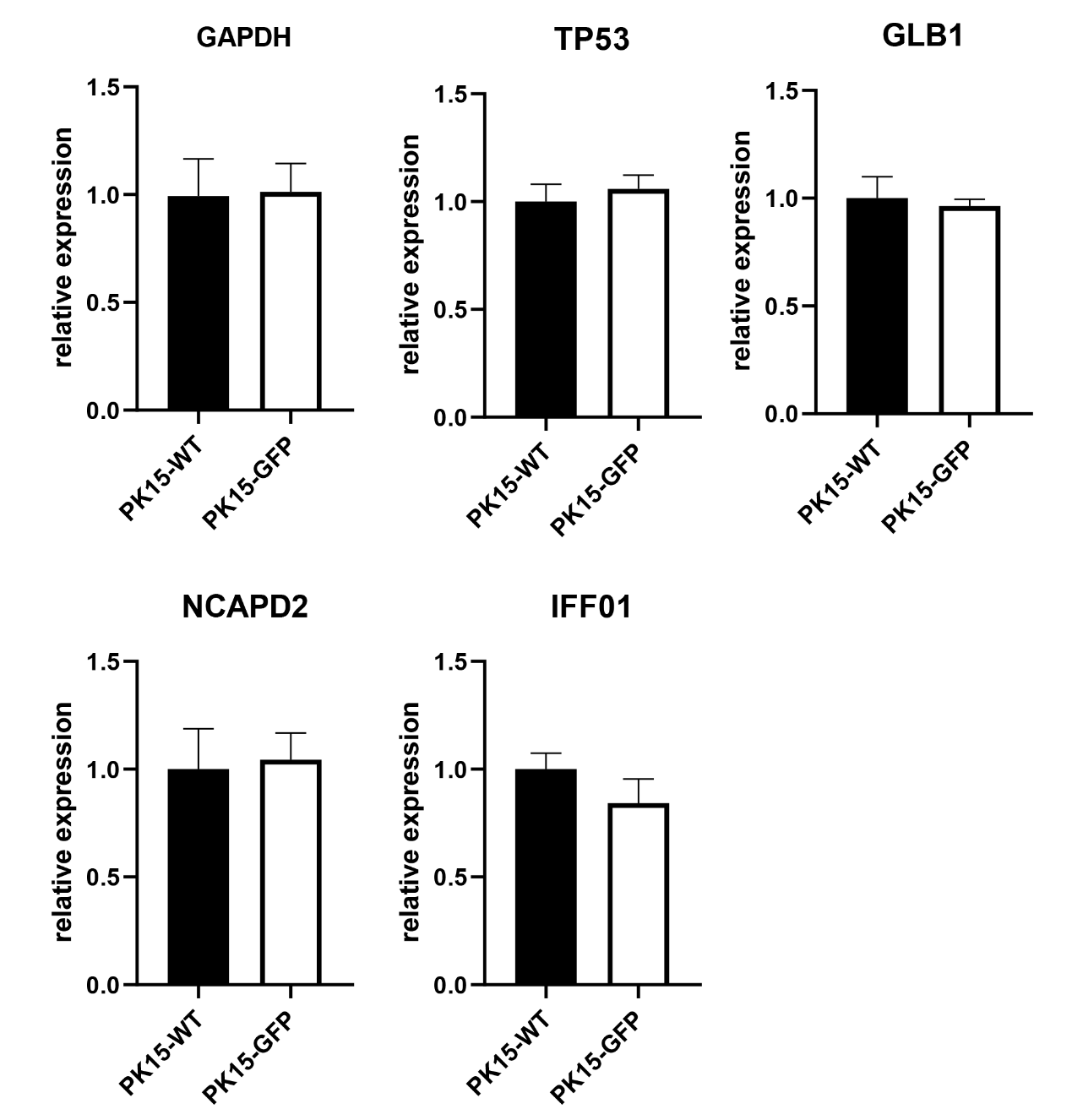
**

**Figure S4.** Validation of the differences at the transcriptional level between GFP knock-in PK15 cells and wild type PK15 cells by QPCR. Five genes (*GAPDH*, *TP53*, *GLB1*, *NCAPD2* and *IFF01*) were picked to performed QPCR assay. PK15-WT, wild type PK15 cells; PK-GFP, GFP knock-in PK15 cells; Error bars represent standard error (SEM) of the mean.

**Table S1.** **Primers used in this study**

| **Primers** | **sequence（5‘-3’）** | **Amplicon** |
| --- | --- | --- |
| **GAPDH-sgR-F** | **caccCATGGTCCACATGGCCTCCA** |  |
| **GAPDH-sgR-R** | **aaacTGGAGGCCATGTGGACCATG** |  |
| **P1** | **CAGGACATCAGAGGAGGGGTA** | **1120bp** |
| **P2** | **CTGAACTTGTGGCCGTTTAC** |  |
| **P3** | **CTGAGCAAAGACCCCAACGA** | **1069bp** |
| **P4** | **ATGGGGAGACGTGACTGAGG** |  |
| **Prediated-OFF-Target1-F** | **AAAAACCAACCAACCAACCA** | **697bp** |
| **Prediated-OFF-Target1-R** | **CTTGGGAGTTTTCCCATTCA** |  |
| **Prediated-OFF-Target2-F** | **CAGTGAGAACGTGGTAGGACC** | **823bp** |
| **Prediated-OFF-Target2-R** | **CAGGATCCAGCCAACAGACA** |  |
| **Prediated-OFF-Target3-F** | **ACTCTCACAAGCAGCTGTCC** | **727bp** |
| **Prediated-OFF-Target3-R** | **CCCCTCATCCACCTTGTCAC** |  |
| **Prediated-OFF-Target4-F** | **AGTCTTCTGCTGTTTCACCACA** | **609bp** |
| **Prediated-OFF-Target4-R** | **CCCACTCCATGCCTGGAAAC** |  |
| **Prediated-OFF-Target5-F** | **TTCTTTGCAGCACCATTCAC** | **700bp** |
| **Prediated-OFF-Target5-R** | **ATGGACACTGGTGTGTTGGA** |  |
| **Prediated-OFF-Target6-F** | **AGCAGCAAACTGGACACTCA** | **680bp** |
| **Prediated-OFF-Target6-R** | **ACCTACCCCAAACAACCTTCT** |  |
| **Prediated-OFF-Target7-F** | **CGGGCCGAGTTGGATATCAG** | **592bp** |
| **Prediated-OFF-Target7-R** | **GATACTGCCACACCCACACT** |  |
| **GAPDH-189bp-F** | **TCGGAGTGAACGGATTTGGC** | 189bp |
| **GAPDH-189bp-R** | **TGACAAGCTTCCCGTTCTCC** |  |
| **GLB1-179bp-F** | **TTCACTATTTCCGCATGCCC** | 179bp |
| **GLB1-179bp-R** | **CAGCTGATGGGCCAGTCTAAT** |  |
| **TP53-135bp-F** | **CGCTTCGAGATGTTCCGAGA** | 135bp |
| **TP53-135bp-R** | **TTTATGGCGGGAGGGAGACT** |  |
| **IFFO1-191bp-F** | **CCTGTTCAAAACCCGGGAGA** | 191bp |
| **IFFO1-191bp-R** | **GAGACTTTCGGTCCCCAGAC** |  |
| **NCAPD2-136bp-F** | **TCTCTTAAAAGCACTGTGAGCC** | 136bp |
| **NCAPD2-136bp-R** | **GATGGACAGTACCTCCTGCAC** |  |
| **ACTB-174bp-F** | **CTCCAGAGCGCAAGTACTCC** | 174bp |
| **ACTB-174bp-R** | **TGCAGGTCCCGAGAGAATGA** |  |
