# Supplementary figures and images for: Identification of Glyceraldehyde-3-Phosphate Dehydrogenase Gene as an Alternative Safe Harbor Locus in Pig Genome
Source: Genes (Basel). 2019 Aug 29;10(9):660. doi: 10.3390/genes10090660 (PMC6770653; doi:10.3390/genes10090660)

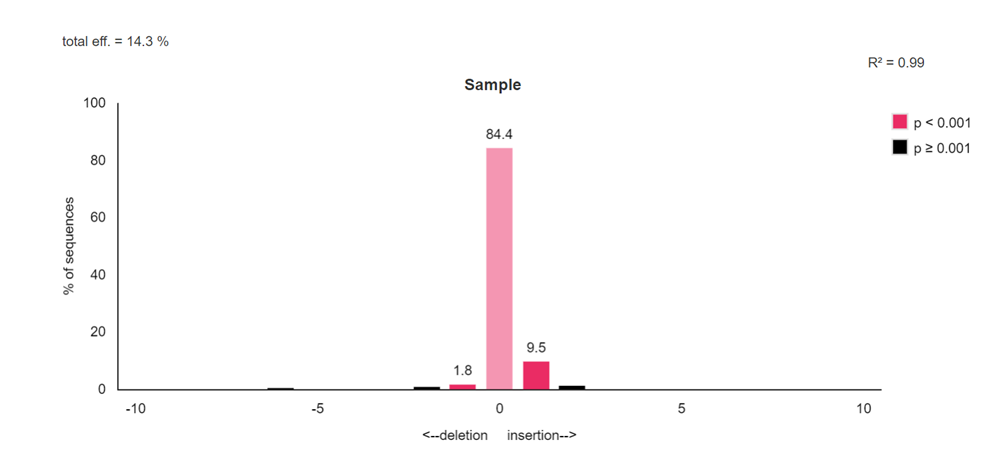

Supplement: Supplementary file 1 [file genes-10-00660-s001.zip › genes-571308-supplementary-revised/Figure S2.tif]

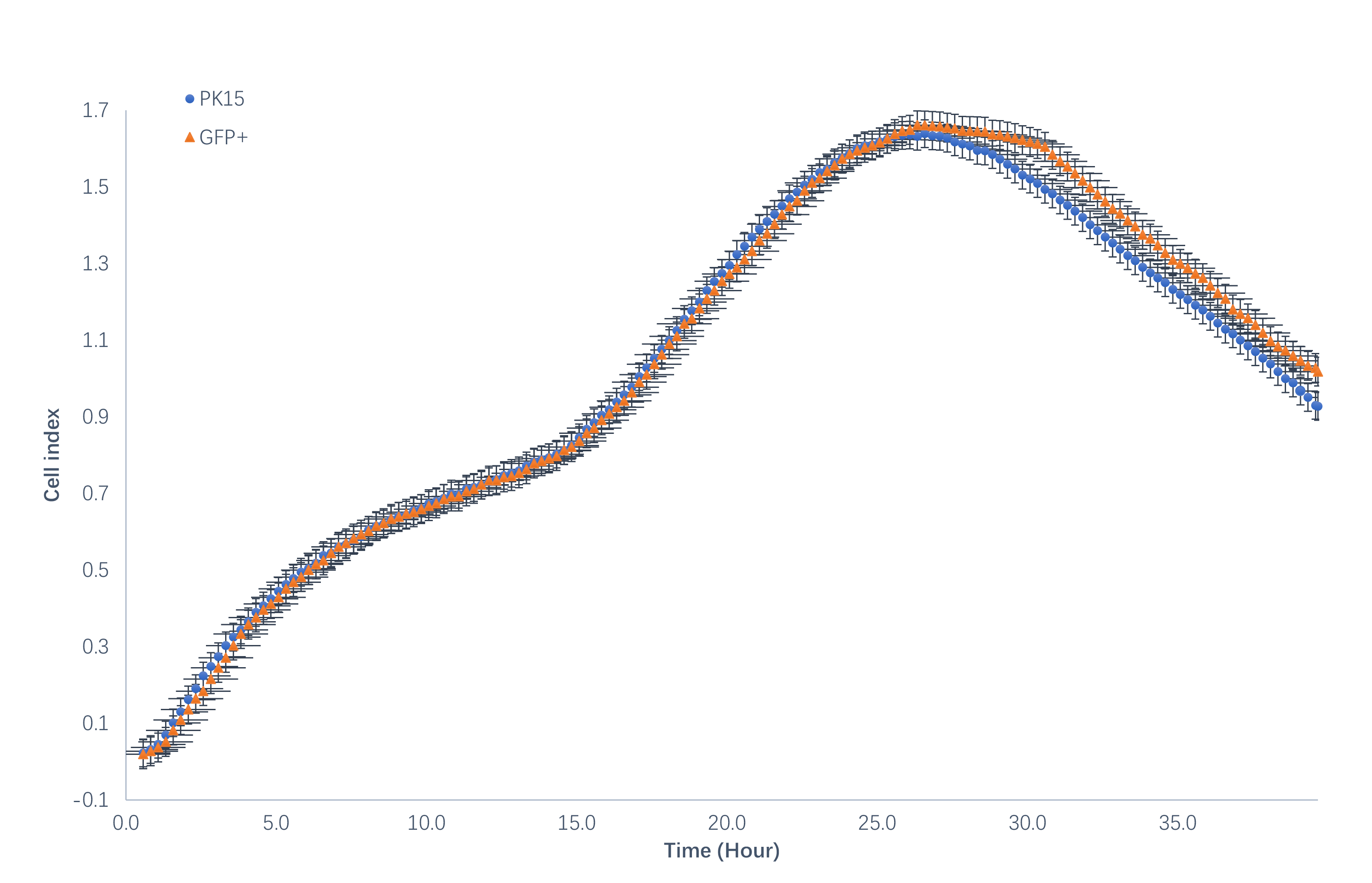

Supplement: Supplementary file 1 [file genes-10-00660-s001.zip › genes-571308-supplementary-revised/Figure S3.tif]

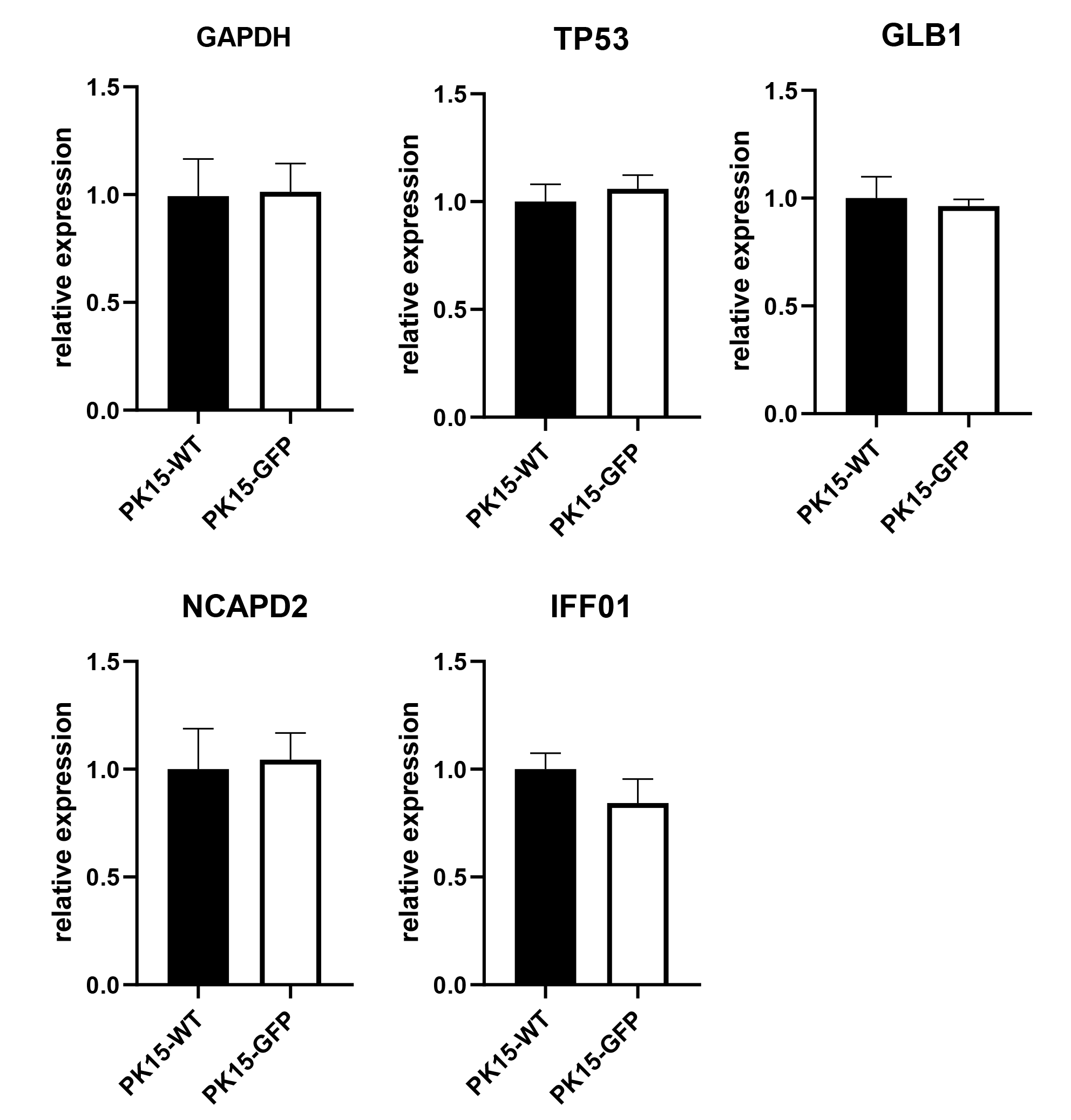

Supplement: Supplementary file 1 [file genes-10-00660-s001.zip › genes-571308-supplementary-revised/Figure S4.tif]
